# Complete Genome Sequence of Strain SDCV/USA/Illinois121/2014, a Porcine Deltacoronavirus from the United States

**DOI:** 10.1128/genomeA.00218-14

**Published:** 2014-04-10

**Authors:** Douglas Marthaler, Yin Jiang, Jim Collins, Kurt Rossow

**Affiliations:** University of Minnesota Veterinary Diagnostic Laboratory, Saint Paul, Minnesota, USA

## Abstract

To investigate the causative agent of swine diarrhea, next-generation sequencing (NGS) was performed on a porcine fecal sample. The NGS reads were assembled, which generated a complete swine *Deltacoronavirus* genome sequence, that of strain SDCV/USA/Illinois121/2014.

## GENOME ANNOUNCEMENT

The International Committee on Taxonomy of Viruses recognizes three coronavirus genera, *Alphacoronavirus*, *Betacoronavirus*, and *Gammacoronavirus*. In addition, the coronavirus genus *Deltacoronavirus* has been proposed (1). Since the discovery of *Deltacoronavirus*, a research investigation was conducted to discover new deltacoronaviruses in a variety of mammalian and avian species. A new swine deltacoronavirus was present in 10% of the porcine samples from China, and two Chinese swine *Deltacoronavirus* complete genome sequences were generated, those of porcine coronavirus (PorCoV) strains HKU15-44 and HKU15-155 (GenBank accession no. JQ065042 and JQ065043, respectively) (2). On 11 February 2014, the Ohio Department of Agriculture announced the presence of a swine deltacoronavirus in the United States. However, the viral pathogenesis and clinical symptoms associated with swine *Deltacoronavirus* infection are still unknown, and the complete U.S. swine *Deltacoronavirus* genome was not released.

The University of Minnesota Veterinary Diagnostic Laboratory received fecal swabs from pigs that had clinical signs of diarrhea but that were negative by real-time PCR (RT-PCR) for other known swine enteric pathogens, including transmissible gastroenteritis virus, porcine epidemic diarrhea virus, and rotaviruses A, B, and C. To further investigate the causative agent of diarrhea, the RNA was extracted from the sample and submitted for next-generation sequencing (NGS), as previously described (3). The NGS reads were *de novo* assembled using SeqMan NGen (version 11; DNAStar, Madison, WI), with the default settings. The contigs were sorted according to length, and a BLAST search was performed on each contig. The first three contigs matched the bacterium *Lactobacillus*, while the fourth contig matched the swine *Deltacoronavirus* PorCoV HKU15-44. The majority of the *de novo* contigs matched bacterium species from *Lactobacillus* and *Enterococcus*. Next, a reference-based assembly was conducted with the PorCoV HKU15-44 strain to generate a complete genome sequence of the swine *Deltacoronavirus*.

The swine *Deltacoronavirus* strain SDCV/USA/Illinois121/2014 (IL121) is 25,405 nucleotides in length, including the partial 5′- and 3′-untranslated regions (UTRs). Strain IL121 has nucleotide percent identities of 99.0% and 99.2% to the previously reported swine *Deltacoronavirus* strains HKU15-44 and HKU15-155, respectively. A single insertion or deletion was observed in the 3′-UTR of strain IL121, which is also present in strain HKU15-155. The spike gene had the lowest nucleotide percent identities of 98.6% and 98.8% to HKU15-155 and HKU15-44, respectively, which correlated to amino acid percent identities of 98.5% and 99.5%, respectively. The envelope gene was the most conserved, with a nucleotide percent identity of 99.6% and an amino acid percent identity of 100% with the previously reported swine *Deltacoronavirus* strains. While nonstructural gene 6 (NS6) of IL121 has high nucleotide percent identities of 99.3% and 99.6% to those of HKU15-155 and HKU15-44, respectively, the amino acid percent identity is only 98.9% to both previously reported swine *Deltacoronavirus* strains.

To our knowledge, this is the first complete genome sequence of a swine deltacoronavirus from the United States. Further in-depth phylogenetic analysis of swine deltacoronaviruses in the United States requires more U.S. and worldwide complete genomes from swine deltacoronaviruses. The release of strain SDCV/USA/Illinois121/2014 should further expedite swine deltacoronavirus research on the genetic diversity, epidemiology, and evolution of swine deltacoronaviruses in the United States.

### Nucleotide sequence accession number.

The genome sequence of the swine *Deltacoronavirus* strain SDCV/USA/Illinois121/2014 was deposited in GenBank with the accession no. KJ481931.
